# Artificial Intelligence in Vascular Surgery: A Literature Review Focusing on Current Applications, Imaging Advances and Future Prospects

**DOI:** 10.3390/jcm15134988

**Published:** 2026-06-26

**Authors:** Areeb Ansari, Nabiha Ansari, Shehzad Zaheer, Usman Khalid, Kristian Bechev, Daniel Markov, Vladimir Aleksiev, Galabin Markov, Elena Poryazova

**Affiliations:** 1 Faculty of Medicine, Medical University of Plovdiv, 4002 Plovdiv, Bulgaria; nabiha.m.ansari@gmail.com (N.A.); shehzadzaheer@hotmail.com (S.Z.); usmankhalid957@gmail.com (U.K.); gabi_markov@abv.bg (G.M.); 2 Department of Anatomy, Histology and Cytology, Faculty of Medicine, Medical University of Plovdiv, 4002 Plovdiv, Bulgaria; kristian_bechev@abv.bg; 3 Neurological Surgery, Pulmed University Hospital, 4000 Plovdiv, Bulgaria; 4 Department of General and Clinical Pathology, Faculty of Medicine, Medical University of Plovdiv, 4002 Plovdiv, Bulgaria; daniel.markov@mu-plovdiv.bg (D.M.); eporiazova@abv.bg (E.P.); 5 Department of Clinical Pathology, University Hospital “Pulmed”, 4002 Plovdiv, Bulgaria; 6 Department of Thoracic Surgery, University Hospital “Kaspela”, 4002 Plovdiv, Bulgaria; vl_alex@abv.bg; 7 Department of Cardiovascular Surgery, Medical University of Plovdiv, 4002 Plovdiv, Bulgaria; 8 Clinical and Experimental Morphology Division, Research Institute at Medical University of Plovdiv, Medical University of Plovdiv, 4002 Plovdiv, Bulgaria

**Keywords:** artificial intelligence, vascular surgery, diagnosis, treatment, imaging, current applications

## Abstract

**Background/Objectives:** Artificial intelligence (AI) is increasingly being integrated into vascular surgery, particularly in diagnostic imaging, perioperative planning, intraoperative guidance, and postoperative surveillance. This literature review evaluates the current applications of artificial intelligence in vascular surgery and endovascular practice, with a particular focus on imaging technologies and their role in improving diagnostic precision, workflow efficiency, and patient outcomes. In addition, the review examines emerging AI applications in operative workflow optimization, endovascular navigation, postoperative surveillance, training platforms, and AI-assisted clinical decision support. **Methods:** A literature review was conducted using PubMed and Scopus with the search terms: (artificial intelligence OR AI OR neural network) AND (vascular surgery) AND (diagnosis OR treatment). Reference lists of included studies were manually screened, and additional recent studies were identified from relevant journals. Articles published in English up to April 2026 were included. Studies were assessed for their applications in vascular diagnostics, plaque characterization, endovascular workflow optimization, and postoperative surveillance. **Results:** AI demonstrated strong diagnostic performance across multiple imaging modalities. Deep learning systems achieved a sensitivity of 91.3% and specificity of 95.2% in peripheral arterial stenosis classification, while plaque characterization models showed accuracies up to 96% and substantial agreement with expert imaging interpretation. AI-assisted operative systems improved procedural efficiency through reductions in operative duration, radiation exposure, and contrast utilization. However, many studies were retrospective, single-center, and based on relatively small cohorts with heterogeneous endpoints. **Conclusions:** AI has significant potential to improve vascular surgical practice through enhanced image interpretation, procedural guidance, and individualized treatment planning. Despite promising outcomes, current evidence remains limited by methodological heterogeneity and insufficient external validation. Prospective multicenter studies and standardized evaluation frameworks are required before widespread clinical implementation can be achieved.

## 1. Introduction

Artificial Intelligence (AI) encompasses a culmination of data analysis, pattern recognition, and outcome prediction. The individuality of these intelligent programs permits for a multifaceted utility in the landscape of patient care [1]. Leveraging the potential of AI in vascular surgery converges on four key applicational branches: vasculo-diagnostics, perioperative medicine, risk stratification, and auto-enhanced prediction [2]. The advancement of personalized medicine has underscored the value of AI in tailoring diagnostics and treatments to individual patient profiles. In vascular surgery, this supports more precise decision-making through intelligent software capable of analyzing complex imaging datasets. Despite its potential, the clinical adoption of AI remains limited, largely due to the lack of formal guidelines from UK professional surgical bodies, which continues to restrict its structured integration into standard practice [3].

Recent evidence suggests that artificial intelligence applications in vascular surgery extend beyond diagnostic imaging alone. A recent scoping review by Powezka et al. highlighted the expanding role of AI across vascular and endovascular practice, including imaging analysis, risk prediction, procedural planning, clinical decision support, and workflow optimization. These findings underscore the broad and evolving impact of AI technologies within vascular surgery and support the need for comprehensive evaluation of both diagnostic and procedural applications [4].

## 2. Materials and Methods

A comprehensive search of the PubMed and Scopus database was conducted, with the keywords: (artificial intelligence OR AI OR neural network) AND (vascular surgery) AND (diagnosis OR treatment). These terms were chosen to capture a wide range of studies relevant to the application of AI in vascular diagnostics and treatment. Additional recent studies were identified through manual screening of relevant publisher journals, relevant to vascular surgery and artificial intelligence. To minimize the risk of omitting significant studies, the reference lists of all included articles were also reviewed manually. Following the electronic database search, additional relevant studies were identified through manual reference-list screening and citation tracking of eligible articles. Records retrieved from the database search and manual screening were combined into a single study library prior to screening. Duplicate records arising from overlap between database results and manually identified studies were removed during an initial deduplication step based on title, author list, publication year, and DOI. The remaining unique records were subsequently screened by title and abstract for eligibility.

Inclusion criteria comprised original research studies, observational studies, retrospective and prospective clinical investigations, technical validation studies, and relevant review articles evaluating artificial intelligence applications within vascular surgery and endovascular practice. Eligible studies included applications involving peripheral arterial disease, aortic aneurysm disease, carotid artery disease, endovascular interventions, vascular imaging, plaque characterization, vascular anomaly assessment, and AI-assisted procedural planning or surveillance. Given the translational relevance of certain imaging technologies, selected coronary and neurovascular studies were included where their methodologies or findings were considered applicable to vascular imaging and endovascular practice. Studies evaluating machine learning, deep learning, radiomics, computer-aided detection, image analysis, predictive modelling, and AI-assisted clinical decision support systems were considered eligible. Technical and preclinical validation studies were included when they demonstrated potential clinical applicability to vascular imaging or intervention.

Exclusion criteria included non-English publications, conference abstracts, editorials, letters to the editor, studies unrelated to vascular or endovascular disease, studies without a clear artificial intelligence component, and publications focused exclusively on non-clinical engineering developments without potential translational relevance to vascular practice. Study selection was performed independently by two reviewers through title, abstract, and full-text screening. Any disagreements regarding study eligibility were resolved through discussion and consensus between the reviewers.

Articles published up to April 2026 were considered eligible, ensuring incorporation of recent evidence relevant to current developments in artificial intelligence within vascular surgery, provided they were available in English. Non-English publications were excluded to maintain consistency in data interpretation and analysis.

**Figure 1:**
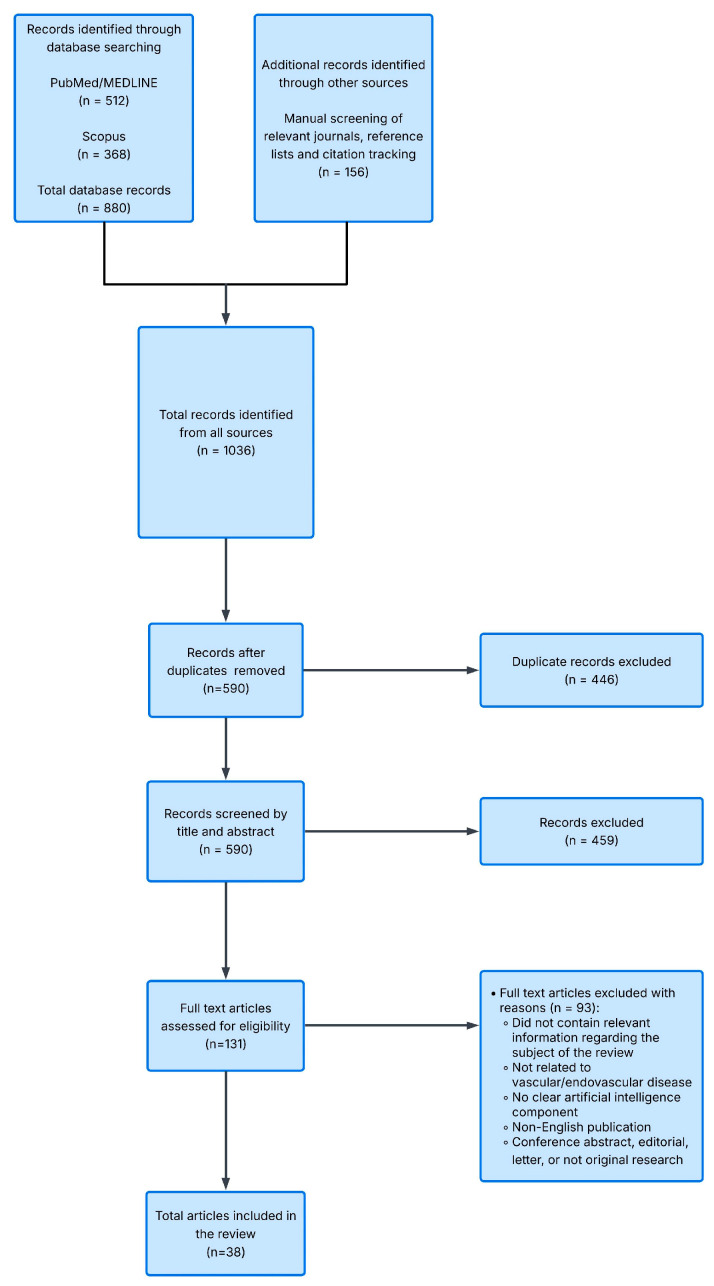
Study selection flow diagram.

The initial search yielded 1036 results. The process for screening, inclusion, and exclusion of articles is outlined in the study selection flow diagram (Figure 1), which details the stepwise methodology used to determine the final set of studies included for review.

## 3. Results

### 3.1. Artificial Intelligence in Peripheral Arterial Disease Assessment

#### Stenosis Detection and Diagnostic Imaging

Patient management is employed on the principle of disease severity, through the evaluation of operative risks, underscoring the necessity for a careful and timely assessment of disease progression. Pressures and waveforms in photoelectric plethysmography (PPG) play an auxiliary role in the ultrasonographic interpretation of peripheral artery disease (PAD). Despite the standardization of duplex carotid ultrasound interpretation by flow velocities, analysis of lower extremity arterial Doppler (LEAD) ultrasound is more nuanced, requiring a multi-level evaluation of both pressures and waveforms for result formulation. With the design of neural networks, enhanced waveform analysis can be performed to better assess the risk of adverse event detection. By capturing nonlinear relationships between variables, the machine learning analysis of the Doppler waveform optimizes performance output through error modification, identifying patients at risk for all-cause death within 5 years with a hazard ratio of 2.44 [5]. Nevertheless, within a clinical setting, the application of machine learning (ML) is limited by the laborious manual segmentation of labelled data to serve as a ground truth, which proves to be a tedious and time-consuming process [6].

Computed tomography angiography (CTA) remains an integral tool for determining intervention strategies by visualizing a roadmap of vascularization, assisting surgeons in the evaluation of vascular anatomy [7]. The heterogeneous presentation of peripheral pathologies poses challenges for artificial networks, with circulatory blockage complicated by artifacts from stents, calcifications, and stenoses [6]. To address this, neural networks must be trained on an abundance of data to optimize disease screening, classification, and workflow. Over 17,000 axial images of the iliac, femoropopliteal, and infrapopliteal artery were fed to a neural network to enhance the quantitative analysis of peripheral stenoses, improving image segmentation, achieving a sensitivity of 91.3% with a specificity of 95.2% respectively when classifying below-knee artery, demonstrating pivotal results in mitigating diagnostic errors and facilitating timely, targeted interventions. To complement this, workload reductions enable plaque detections that may have otherwise gone unnoticed [8].

### 3.2. Artificial Intelligence in Aortic Disease and Endovascular Intervention

#### 3.2.1. EVAR Planning and Intraoperative Guidance

**Figure 2:**
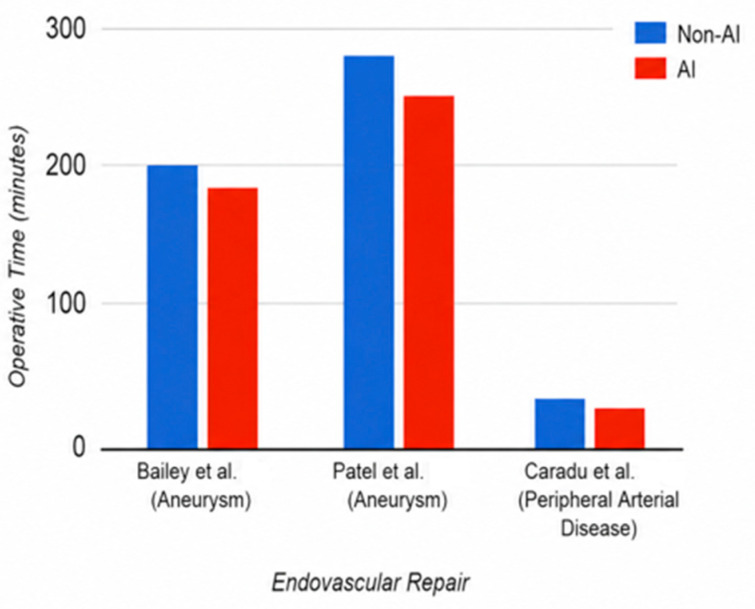
Comparison of the effects of AI on operative time during endovascular repair across different studies [9,10,11].

**Figure 3:**
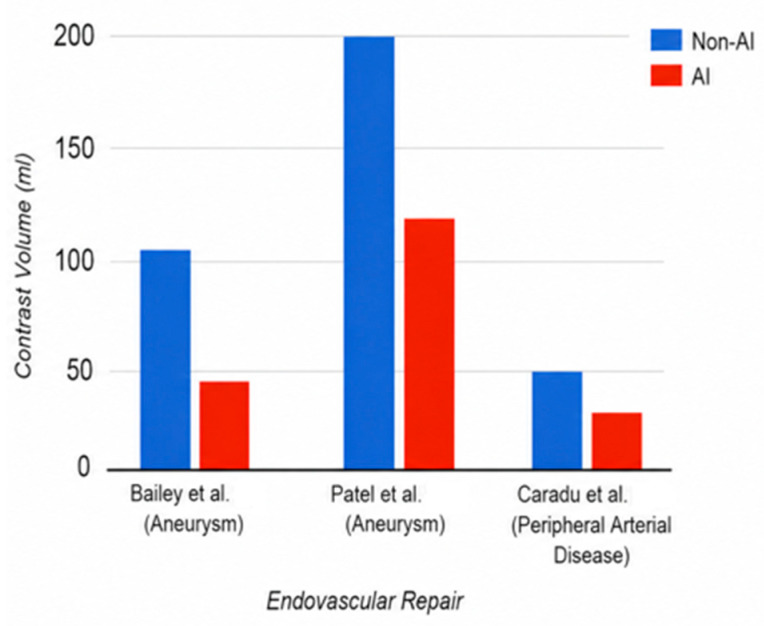
Comparison of the effects of AI on contrast volume used during endovascular repair across different studies [9,10,11].

A leap in advancements has landed AI directly into the operating theatre, collaborating with surgeons in endovascular navigation and repair. These complex models are designed to amplify the selection of stents or medical devices, streamline the process of navigating through blood vessels, and improve the accuracy of stent positioning. A cloud-based fusion platform, Cydar EV Intelligent Maps enhances surgical efficiency and safety by guiding surgeons through endovascular aneurysm repair (EVAR). With aortic volume rendering and overlay mapping, the platform streamlines vessel pre-cannulation, device orientation, and deployment, reducing the procedural time and need for iodinated contrast. With a reduction in radiation exposure, contrast volume, and fluoroscopy times during endovascular procedures, patient and physician protection was enhanced [9,10]. Complexities create intense scenarios requiring optimal planning and procedural practice. Endonaut, a similar navigation station, creates a two-dimensional panorama of the pathologically impacted peripheral arterial system from a sequence of fluoroscopic images. Integration permits image synchronization with the angiography panorama, creating the foundation of the image fusion system [11]. The impact of Artificial Intelligence on operative times and contrast volumes is independently depicted below in Figure 2 and Figure 3, showcasing the efficiency gains when utilizing an AI-based diagnostic panel in three studies [9,10,11].

#### 3.2.2. Endoleak Detection and Postoperative Surveillance

Given that Endoleaks can affect up to 20% of patients following endovascular aneurysm repair (EVAR), continuous surveillance of postoperative outcomes is crucial for enabling timely intervention strategies [12]. The Endo-Detecto algorithm represents a significant advancement in detecting post-procedural complications through meticulous data integration, incorporating factors such as endoleak detection, maximum abdominal aortic aneurysm (AAA) diameter, AAA volume, and endoleak volume. This thorough approach enables the binary classification component of the pipeline to achieve impressive results, accurately classifying 88% of endoleaks in CT scans [13]. The integration of algorithms designed to minimize error susceptibility while optimizing time efficiency provides a compelling foundation for their clinical application.

The EndoLeak Augmentor, an advanced successor of these models, demonstrated superior performance compared to the algorithm evaluated in Hahn’s study, highlighting the critical role of diverse and extensive datasets in augmenting machine learning capabilities. This underscores the need for high-quality data to refine model training and validation processes further, driving improvements in detection accuracy. Ultimately, the consistency, efficiency, and precision of AI-driven solutions are revolutionizing the detection of vascular complications, setting new benchmarks for diagnostic accuracy in clinical settings [14]. See Table 1.

#### 3.2.3. Workflow Optimization and Clinical Decision Support

Beyond imaging enhancement, artificial intelligence is increasingly contributing to operative workflow optimization and perioperative decision support within vascular surgery. A recent systematic review evaluating 817 initially identified studies, of which eight met final eligibility criteria, demonstrated that AI applications were most utilized during the preoperative phase for anatomical assessment, operative planning, and risk prediction. Among the reviewed studies, AI-based predictive models achieved strong technical performance, including an AUROC of 0.90 for carotid endarterectomy outcome prediction, intraclass correlation coefficients (ICCs) of 0.94 for EVAR volumetric analysis, and greater than 80% predictive accuracy for arteriovenous fistula maturation. Intraoperative AI-assisted systems further demonstrated improvements in workflow efficiency through reductions in operative duration, radiation exposure, and iodinated contrast utilization during endovascular procedures. Despite these promising outcomes, the review emphasized that six of the eight included studies were retrospective in design, with substantial heterogeneity and limited external validation, restricting widespread clinical implementation at present [15].

Translational reviews have further emphasized the expanding role of computer-aided artificial intelligence systems in endovascular interventions. A 2026 review published in Bioengineering evaluated AI-CAD applications across neurovascular, coronary, aortic, and peripheral endovascular domains using literature from 2020–2026 identified through PubMed, Google Scholar, and IEEE Xplore databases. The review highlighted that automated large-vessel occlusion detection systems were capable of improving treatment duration metrics in acute stroke pathways, while deep learning assisted endoleak detection models demonstrated increasing utility during endovascular aneurysm repair (EVAR). Furthermore, the authors emphasized that AI-assisted angiographic segmentation systems improved reproducibility compared with conventional manual measurements in both coronary and peripheral vascular imaging. Despite these advances, the review identified major translational barriers including acquisition variability, metallic artifact interference, limited multicenter validation, and substantial heterogeneity in performance reporting across studies. The authors ultimately concluded that rigorous external validation, lesion-specific performance assessment, and workflow-centered outcome evaluation remain essential before widespread integration of AI-CAD systems into routine endovascular practice [16].

This article further reviewed AI-CAD systems across coronary, aortic, neurovascular, and peripheral interventions, demonstrating their value in lesion detection, endoleak surveillance, and procedural decision support. In acute neurovascular applications, automated AI triage was highlighted as particularly significant given that approximately 1.9 million neurons are lost per minute during untreated large-vessel occlusion. However, despite improving workflow efficiency and reducing operator dependency, most reported systems were validated using retrospective single-center datasets with limited multicenter external validation [16].

### 3.3. Artificial Intelligence in Plaque Characterization and Intravascular Imaging

#### 3.3.1. Optical Coherence Tomography (OCT)

The integration of artificial intelligence (AI) into vascular imaging has markedly transformed plaque characterization, offering improved speed, reproducibility, and diagnostic precision across multiple imaging modalities. Recent developments highlight the growing role of deep learning models in automating plaque detection, classification, and quantification, with significant implications for risk stratification and procedural planning in coronary artery disease.

Among imaging modalities, optical coherence tomography (OCT) has particularly benefited from AI enhancement. A large multicenter study developed and externally validated a deep learning framework trained on 10,517 intravascular OCT (IVOCT) cross-sections, achieving excellent performance across plaque types. The model attained Dice scores of 0.906 for fibrous plaques, 0.848 for calcifications, and 0.772 for lipidic plaques, alongside strong agreement with manual quantitative measures (R^2^ = 0.98). Diagnostic accuracy for plaque type classification was 86.6%, with especially high performance for fibrous (97.6%), lipidic (90.5%), and calcified (88.5%) plaques. With a median processing time of 21.4 s per pullback and consistent performance across different imaging platforms, this approach presents a practical tool for enhancing objectivity in clinical decision-making. However, the study remained dependent on highly curated OCT imaging datasets with limited external validation, potentially restricting generalizability across broader vascular imaging populations [16].

Further refining OCT-based plaque analysis, another study focused specifically on deep learning detection of calcified plaques. Among 72 anatomical slices, 95% of plaques identified by AI were validated via OCT-derived optical properties. Substantial agreement with greyscale IVUS (kappa 0.69), IVUS–virtual histology (0.60), and echogenicity (0.60) was observed, improving further after artifact exclusion (kappa up to 0.77). The AI-derived calcium arc measurements showed moderate to good correlation with ultrasound-based measures (ICCa 0.81 for greyscale IVUS), reinforcing the complementary role of AI-enhanced OCT in multimodal plaque characterization. Despite strong multimodal imaging performance, further prospective validation is required to determine whether combined IVUS-OCT AI systems can be reliably integrated into routine vascular clinical workflows [17].

Complementing these efforts, a hybrid modelling strategy combining deep learning convolutional features with hand-crafted lumen morphology features further advanced automated plaque characterization in IVOCT imaging. Using a dataset of 6556 images, the hybrid model achieved high sensitivity and specificity for fibro-lipidic plaques (84.8%/97.8%) and fibrocalcific plaques (91.4%/95.7%). The inclusion of morphology features significantly improved performance compared to deep learning alone (*p* < 0.05), while active learning and expert relabeling particularly enhanced fibrocalcific plaque classification. These developments demonstrate the value of hybrid approaches in improving classification accuracy and clinical relevance. Nevertheless, the reliance on controlled OCT datasets and limited external testing may reduce the robustness and broader reproducibility of fully automated plaque characterization systems [18].

#### 3.3.2. Coronary Computed Tomography Angiography (CCTA)

Beyond OCT, substantial progress has been made in coronary computed tomography angiography (CCTA) analysis. A deep learning model developed using 887 patients and externally validated on 221 patients demonstrated strong agreement with expert readers for plaque quantification and stenosis evaluation. Intraclass correlation coefficients were 0.876 for total plaque volume, 0.869 for noncalcified plaque volume, and 0.850 for percent diameter stenosis. Against IVUS benchmarks, the AI achieved ICCs of 0.945 for total plaque volume and 0.853 for plaque burden. Importantly, analysis times were reduced to approximately 20 s per patient compared to 25–30 min for manual expert readings, emphasizing the efficiency gains achievable with AI integration. Although external validation strengthened the study methodology, differences between CTA-derived and IVUS-derived plaque measurements may still introduce variability in cross-modality interpretation and endpoint standardization [19].

Expanding on this, a recurrent convolutional neural network (RCNN) was developed for simultaneous plaque and stenosis assessment from CCTA images in 163 patients. The model achieved an accuracy of 0.77 for plaque characterization and 0.80 for stenosis detection, with improved performance (0.85 accuracy) when simplified to binary plaque detection. Anatomically significant stenosis (≥50% narrowing) was detected with a segment-level accuracy of 0.94, demonstrating the potential of advanced deep learning architectures such as recurrent networks and multitask learning in vascular imaging. However, the retrospective nature of the imaging dataset and rapid evolution of contemporary deep learning architectures may limit the broader applicability of these findings to current clinical AI systems [20].

#### 3.3.3. Intravascular Ultrasound (IVUS)

Within intravascular ultrasound (IVUS) imaging, a separate study applied deep learning techniques to 598 patients to automate plaque characterization, specifically targeting attenuation and calcification. The AI model achieved frame-level accuracies of 93% for attenuated plaque and 96% for calcification, with strong correlations to human expert measurements (r = 0.89–0.95). With a processing time of 0.05 s per frame, the system demonstrates feasibility for near-time clinical integration, providing an objective and efficient alternative to the traditional labor-intensive manual interpretation of IVUS images. Nonetheless, the operator-dependent nature of IVUS acquisition and potential variability in plaque annotation may limit reproducibility across different vascular imaging centers [21].

#### 3.3.4. Plaque Vulnerability Assessment and Risk Stratification

Importantly, AI has not only improved imaging efficiency but has also enhanced prognostic capabilities. In a multicenter study involving 1791 patients undergoing OCT, a DenseNet-121-based model was trained on 44,947 images to distinguish between normal segments, stable plaques, and vulnerable plaques. The AI model showed excellent diagnostic performance, and the presence of vulnerable plaques identified by AI was significantly associated with higher clinical event rates (log-rank *p* < 0.001). Vulnerable plaque identification remained an independent predictor of events on multivariate analysis (*p* = 0.047 segment-specific; *p* < 0.001 overall), highlighting the potential role of AI in refining risk stratification and guiding personalized management strategies. Although the integration of OCT-derived plaque vulnerability assessment with clinical outcomes is promising, variability in outcome definitions and longitudinal follow-up may limit comparability across studies [22].

**Figure 4:**
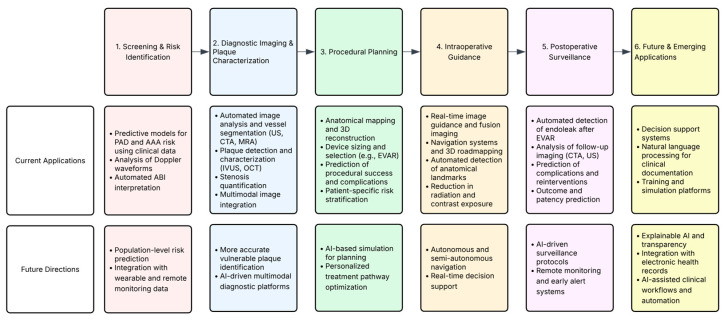
Central AI workflow overview in vascular surgery.

Together, these studies underscore AI’s transformative impact across multiple vascular imaging modalities. By enhancing the speed, accuracy, and reproducibility of plaque characterization, AI is poised to become an integral component of clinical workflows, improving both diagnostic precision and patient outcomes in vascular disease. See Table 2 and Figure 4.

## 4. Discussion

### 4.1. Distinguishing Concepts

Artificial intelligence (AI) refers to computational systems capable of performing tasks that typically require human intelligence, including machine learning (ML) and deep learning (DL) algorithms used for prediction, classification, segmentation, and decision support. Radiomics refers to the extraction and analysis of quantitative imaging features for diagnostic or prognostic modelling, often in conjunction with ML techniques. Computer-aided detection (CAD) systems are designed to assist clinicians in identifying imaging abnormalities but may not necessarily employ advanced AI methodologies. In contrast, image-fusion technologies, augmented reality platforms, and endovascular navigation systems primarily facilitate image visualization, procedural guidance, and workflow enhancement and should not be considered standalone AI applications unless they incorporate ML- or DL-based analytical components. Throughout this review, these technologies are discussed separately to distinguish AI-driven diagnostic tools from image-guidance and procedural support systems.

### 4.2. Infancy in AI—Issues and Future Considerations

Despite the promise of AI in modern medicine, progress is hindered by limitations in data volume and diversity. Without standardized tools to segment global demographic data, machine learning models risk producing outputs that do not represent entire populations [24]. This is crucial in personalized medicine, where AI algorithms may be skewed by over-reliance on data from specific regions, ethnicities, or socioeconomic groups. The lack of diverse datasets could exacerbate health disparities. The ethical challenges around security, privacy, and regulation demand transparency and reliability. A notable example is the 2022 controversy involving the University of California, which faced backlash for sharing patient data with Google to develop healthcare algorithms. This partnership sparked concerns over potential re-identification of patients, despite claims of anonymization. These incidents underline the need for clear guidelines on data sharing and anonymization practices [25,26].

The interpretability of artificial intelligence systems remains another major barrier to widespread clinical implementation. Recent work published in Bioengineering proposed a structured explainability framework for AI-enabled medical imaging systems based on four key assessment domains: consistency, plausibility, fidelity, and clinical usefulness. The framework was designed to evaluate the reliability and transparency of explainable AI (XAI) features within diagnostic imaging platforms, particularly in high-risk clinical settings where algorithmic decision-making directly influences patient management. Using breast lesion detection on synthetic mammography as a case study, the authors demonstrated how explanation heatmaps generated through Ablation-CAM and Eigen-CAM could be quantitatively assessed for alignment with model reasoning and diagnostic relevance. The study further emphasized that standardized explainability metrics may improve clinician trust, reproducibility, and medico-legal accountability, supporting safer integration of AI-assisted diagnostic systems into routine clinical workflows [27].

When algorithms reflect societal inequalities, they risk reinforcing existing disparities in patient care, leading to poorer outcomes for already marginalized populations. This is particularly concerning for clinical decision-making tools, where biased outputs can result in misdiagnosis or unequal access to treatments. Addressing this bias is essential to ensure that technological advancements in medicine benefit all groups equitably. One of the most effective ways to minimize profound algorithmic bias is through large-scale, demographically diverse data training. However, many current AI models rely on centralized data pools drawn from limited geographic or institutional sources. This introduces a significant epidemiological limitation, making it difficult to train models that are generalizable across global populations and further restricting equitable access to AI-driven healthcare solutions [14].

Only recently, in April of 2024, has the MHRA outlined its strategic approach to transforming the role of AI in the UK healthcare sector by 2030, but given the early adoption of such principles, it creates ambiguity in decision-making and implementation until continuous use is integrated within the health sector [28]. Whereas in the United States, the development of the “Artificial Intelligence and Machine Learning (AI/ML) Software as a Medical Device Action Plan.” (https://www.fda.gov/medical-devices/software-medical-device-samd/artificial-intelligence-software-medical-device accessed on 28 March 2026), since 2021 has outlined a framework designed to guide AI and ML companies while ensuring that their software, classified as medical devices, remains safe, effective and continuously updated [29].

### 4.3. Multimodal Imaging and Workflow Optimization with Artificial Intelligence

Another emerging area in vascular diagnostics is the integration of multimodal imaging with artificial intelligence to improve both anatomical and functional assessment of vascular disease. Traditional imaging techniques such as duplex ultrasonography, computed tomography angiography (CTA), magnetic resonance angiography (MRA), and positron emission tomography (PET) are often interpreted independently, despite each modality offering complementary diagnostic information. AI-driven multimodal fusion models have demonstrated the capacity to integrate these datasets into a single analytical framework, allowing for more comprehensive vascular evaluation [30]. This has shown promise in the assessment of carotid artery disease, where combining plaque morphology, hemodynamic flow characteristics, and inflammatory imaging biomarkers may improve the identification of vulnerable plaques at higher risk of rupture or embolization [31]. Furthermore, radiomics-based AI systems are increasingly capable of extracting subtle quantitative imaging features that are imperceptible to the human eye, enabling earlier disease detection and more accurate prediction of disease progression [24,32].

In addition to diagnostic enhancement, AI-assisted imaging is beginning to reshape workflow efficiency within vascular departments. Automated image interpretation systems have the potential to reduce reporting times, prioritize urgent scans, and decrease interobserver variability among clinicians [24]. Deep learning algorithms capable of real-time image analysis may also facilitate remote diagnostics and telemedicine applications, expanding specialist vascular assessment to underserved or geographically isolated populations. Despite these advantages, concerns remain regarding algorithm transparency, and medico-legal accountability [33]. For AI systems to achieve widespread clinical adoption, future research must focus not only on improving diagnostic accuracy but also on ensuring interpretability, external validation, and integration into existing clinical pathways. Continued collaboration between vascular surgeons, radiologists, engineers, and regulatory bodies will therefore be essential in translating AI innovations into safe and effective routine clinical practice.

A review further backs this idea by highlighting the role of artificial intelligence in vascular training, demonstrating that AI-assisted extended reality platforms improved technical skill acquisition, reduced procedural errors, and enabled patient-specific rehearsal across carotid artery stenting, EVAR, ruptured abdominal aortic aneurysm scenarios, and peripheral interventions. These systems also allowed objective performance benchmarking through automated error recognition and real-time feedback, although the evidence remained largely simulation-based without long-term clinical outcome validation [34].

More recently, multimodal AI systems integrating imaging interpretation with evidence-based clinical reasoning have demonstrated promising performance in vascular diagnostics. A 2026 study published in Diagnostics introduced the HevaDx framework, an agentic AI system designed for the diagnosis and treatment recommendation of vascular anomalies. Utilizing a large-scale vascular anomaly dataset alongside retrieval-augmented generation (RAG) reasoning, the system achieved a top 3 diagnostic accuracy of 94.8% with a treatment recommendation accuracy of 83.3%. The authors highlighted that separating visual feature extraction from clinical reasoning improved transparency and reduced the “reasoning gap” commonly observed in conventional end-to-end multimodal language models. These findings further support the growing role of multimodal AI frameworks in improving diagnostic precision and individualized treatment planning within vascular medicine [35].

### 4.4. Methodological Quality and Clinical Readiness of Current AI Studies

Despite the promising performance reported across many artificial intelligence applications in vascular surgery, several methodological limitations remain. A substantial proportion of the studies included in this review were retrospective in design and conducted within single institutions, potentially limiting generalizability to broader patient populations. Dataset sizes varied considerably between studies, ranging from small proof-of-concept investigations to larger multicenter cohorts, resulting in significant heterogeneity in model development and validation approaches.

External validation was inconsistently performed, with many studies relying solely on internal datasets for model training and testing. The absence of independent validation cohorts raises concerns regarding overfitting and the reproducibility of reported performance metrics when applied to different patient populations, imaging protocols, or healthcare systems. Furthermore, variability in imaging acquisition techniques, annotation methods, and outcome definitions complicates direct comparison between studies.

Several studies demonstrated excellent technical performance in image segmentation, lesion detection, and classification tasks; however, relatively few evaluated the impact of AI implementation on clinically meaningful outcomes, workflow efficiency, decision-making, or patient prognosis. Consequently, although many AI applications show considerable promise, the majority remain at an early stage of clinical translation.

Future research should prioritize prospective multicenter validation studies, standardized reporting frameworks, transparent model development, external validation across diverse populations, and assessment of real-world clinical effectiveness before widespread adoption into vascular surgical practice can be recommended.

### 4.5. Sex-Specific Considerations and Algorithmic Fairness

An important consideration in the development and implementation of artificial intelligence within vascular surgery is the potential impact of sex-specific differences and algorithmic bias. Significant differences between male and female patients have been reported across multiple vascular pathologies, including variations in vascular anatomy, access vessel caliber, aortic morphology, eligibility for endovascular aneurysm repair (EVAR), perioperative complications, and long-term clinical outcomes. These differences may influence treatment selection, procedural complexity, and postoperative outcomes, highlighting the importance of sex-specific considerations in vascular surgical practice.

AI-driven imaging analysis and predictive modelling may offer opportunities to reduce these disparities by facilitating more personalized risk stratification, procedural planning, and outcome prediction based on patient-specific anatomical characteristics. However, the benefits of AI are dependent upon the quality and representativeness of the datasets used for model development. Models trained on unbalanced datasets that underrepresent female patients may inadvertently perpetuate or amplify existing healthcare disparities, resulting in reduced accuracy and generalizability across patient populations. Consequently, future AI studies should prioritize diverse and representative datasets, transparent reporting of patient demographics, subgroup performance analyses, and external validation across different populations to ensure equitable and clinically applicable AI systems in vascular surgery [36,37].

### 4.6. Practical Barriers to Clinical Implementation

Despite the considerable promise demonstrated by artificial intelligence applications in vascular surgery, several barriers continue to limit widespread clinical adoption. One of the most significant challenges is the lack of robust external validation, as many models are developed and tested using data from a single institution or a limited number of centres. Differences in patient demographics, imaging protocols, equipment, and clinical practices may reduce model performance when applied to new populations. Furthermore, dataset heterogeneity remains a major concern, particularly in vascular imaging, where variations in image acquisition, annotation standards, and outcome definitions can affect model reliability and reproducibility.

Model explainability represents another important consideration. Many deep learning algorithms function as “black-box” systems, making it difficult for clinicians to understand the rationale behind specific predictions or recommendations. Improving transparency and interpretability will be essential to facilitate clinician trust and support safe clinical decision-making. In addition, AI systems require ongoing monitoring and updating to maintain performance as clinical practices, imaging technologies, and patient populations evolve over time.

Several regulatory, ethical, and legal challenges must also be addressed before routine implementation can occur. Regulatory approval pathways for AI-based medical devices continue to evolve, particularly for adaptive models capable of updating after deployment. Questions regarding medico-legal accountability remain unresolved, especially in situations where AI-generated recommendations contribute to diagnostic or therapeutic decisions. Cybersecurity and data privacy considerations are equally important given the large volumes of sensitive clinical and imaging data required for model development and deployment.

Successful integration of AI into vascular surgical practice will also depend on seamless incorporation into existing clinical workflows. AI systems should be designed to augment rather than replace clinician expertise, providing decision support while maintaining appropriate physician oversight. Future research should focus not only on improving technical performance but also on demonstrating real-world clinical utility, workflow integration, cost-effectiveness, and patient-centred outcomes before widespread adoption can be recommended [38].

## 5. Conclusions

Medicine remains a constantly evolving field, and artificial intelligence is increasingly driving that progress. In vascular surgery, AI is shaping the future of patient care through a growing number of applications ranging from diagnostic image segmentation to intraoperative navigation tools, perioperative planning, and postoperative surveillance. These systems are not limited to one function but offer a broad spectrum of utilities, supporting more accurate diagnoses, improved risk stratification, enhanced workflow efficiency, and greater surgical precision.

While the potential of AI is clear, its clinical integration remains limited. The absence of defined roles and formal guidelines continues to slow adoption in everyday vascular practice, while much of the current evidence remains derived from retrospective single-center studies using relatively small cohorts and heterogeneous endpoints. This limits broader generalizability and makes direct comparison between studies difficult. There are still important questions surrounding reliability, standardization, and how these technologies can be safely integrated into existing healthcare systems.

Even so, AI’s role in personalizing medicine is becoming increasingly apparent. Recent developments in multimodal reasoning systems, predictive analytics, and computer-assisted endovascular platforms further demonstrate its potential to support individualized treatment planning and operative decision-making. As technology matures and gains wider clinical trust, prospective multicenter validation and transparent algorithmic frameworks will be essential for implementation. Despite current limitations, AI is on a clear path toward becoming an integral tool in improving outcomes and shaping the future of vascular surgery.

## Figures and Tables

**Table 1:** Summary and comparison of 2 EndoLeak detection Algorithms.

Algorithm	Year	Key Findings	Limitations
Endo-Detector [13]	2020	Best model for binary endoleak detection achieved: AUC of 0.94 ± 0.03. Localization portion of the network predicted the region of interest containing the AAA in 99% of cases. Average absolute volume error: 1.2 ± 1.9 mL.	Single-center study without cross-validation from larger CTA studies. Selective training of models with certain ‘edge’ cases did not select such as CTs with prior embolizations, further limiting the broad use of the algorithm for a varied patient dataset. Does not distinguish endoleak type or thrombus characteristics Further data-training and code refinement to predict source and origin of endoleak.
EndoLeak Augmentor [14]	2020	Machine learning model performance: Accuracy: 95%, Precision: 90%, Recall: 100%, AUC: 0.99. Patient-level prediction is based on an ensemble of individual CT slice predictions. Machine learning model performance: Accuracy: 95%, Precision: 90%, Recall: 100%, AUC: 0.99.	Limited generalizability of the model due to data being pulled from one medical center. The model is limited to the detection of endoleaks with a failure to interpret endoleak type and aneurysm size.

**Table 2:** Comparing Imaging Modality, AI Algorithm Type, Main Results/Conclusions and Limitations in different Articles.

Study	Imaging Modality	AI Algorithm Type	Main Results/Conclusions	Limitations
Chu et al., 2021 [17]	Intravascular Optical Coherence Tomography (IVOCT)	Deep convolutional network	86.6% overall accuracy. Fast and reliable performance across different OCT systems.	Potential overfitting risk.
Huang et al., 2022 [18]	Optical Coherence Tomography (OCT) and Intravascular Ultrasound (IVUS)	Deep Learning	95% confirmation rate. Strong concordance with ultrasound imaging.	Early-stage validation. Limited clinical translation.
Lee et al., 2020 [19]	Intravascular Optical Coherence Tomography (IVOCT)	Hybrid approach combining Convolutional Neural Network (CNN) and hand-crafted features	High sensitivity (84.8%) and specificity (97.8%) for fibro-lipidic plaques. Better than deep learning alone.	Limited external testing. Reproducibility concerns.
Lin et al., 2021 [20]	Computed Tomography Angiography (CTA) and Intravascular Ultrasound (IVUS)	Deep Learning (Convolutional Neural Network (CNN))	Reduced analysis time to 20 s per patient.	Cross-modality variability. Endpoint heterogeneity.
Zreik et al., 2018 [21]	Coronary Computed Tomography Angiography (CCTA)	Multi-task Recurrent Convolutional Neural Network (RCNN)	0.77 accuracy for plaque, 0.80 for stenosis. 94% accuracy for significant stenosis detection.	Retrospective dataset.
Cho et al., 2021 [22]	Intravascular Ultrasound (IVUS)	Deep learning	93% accuracy for attenuation, 96% for calcification. Rapid analysis (0.05 s/frame) suitable for real-time use.	Limited multicenter validation.
Niioka et al., 2022 [23]	Optical Coherence Tomography (OCT)	Deep learning-based Convolutional Neural Network (CNN) (DenseNet-121)	Vulnerable plaques linked to higher clinical event rates Assists in patient risk stratification.	Variable clinical endpoints.

## Data Availability

No new data were created or analyzed in this study. Data sharing is not applicable to this article.

## Abbreviations

The following abbreviations are used in this manuscript:

AI	Artificial Intelligence
ML	Machine Learning
CT	Computed Tomography
AAA	Abdominal Aortic Aneurysm
CTA	Computed Tomography Angiography
AUC	Area Under the Curve (AUC)
EVAR	Endovascular Aneurysm Repair (EVAR)
AUROC	Area Under the Receiver Operating Characteristic Curve
AI-CAD	Artificial Intelligence Computer-Aided Detection
OCT	Optical Coherence Tomography
IVOCT	Intravascular Optical Coherence Tomography
CCTA	Coronary Computed Tomography Angiography
RCNN	Recurrent Convolutional Neural Network
Ablation-CAM	Ablation Class Activation Mapping
PPG	Photoelectric Plethysmography
IVUS	Intravascular Ultrasound
XAI	Explainable AI
MRA	Magnetic Resonance Angiography
RAG	Retrieval-Augmented Generation
